# Does interprofessional collaboration between care levels improve following the creation of an integrated delivery organisation? The Bidasoa case in the Basque Country

**DOI:** 10.5334/ijic.1118

**Published:** 2013-09-20

**Authors:** Roberto Nuño-Solinís, Iñaki Berraondo Zabalegui, Leticia San Martín Rodríguez, Regina Sauto Arce, Marie-Pierre Gagnon

**Affiliations:** O+Berri, Basque Institute for Healthcare Innovation, Sondika, Spain; Bidasoa Integrated Healthcare Organisation, Hondarribia, Spain; Nursing Professional Development and Training, Hospital University of Navarra, Pamplona, Spain; O+Berri, Basque Institute for Healthcare Innovation, Sondika, Spain; Faculty of Nursing, Université Laval, Québec, Canada; Basque Country, Spain

**Keywords:** health services research, delivery of health care, integration, interprofessional relations, collaboration, questionnaires

## Abstract

**Introduction:**

This article explores the impact of the creation of a new integrated delivery organisation on the evolution of interprofessional collaboration between primary and secondary care levels. In particular, the case of the Bidasoa Integrated Healthcare Organisation is analysed.

**Theory and methods:**

The evolution of interprofessional collaboration is measured through a validated Spanish questionnaire, with 10 items and a 5-point Likert scale, based on the D'Amour's model of collaboration [20]. The final sample included 146 observations (doctors and nurses).

**Results:**

The questionnaire identified a significant improvement on the mean scores for interprofessional collaboration of 0.57 points before and after the intervention. A significant improvement was also found in the two dimensions of the measure of interprofessional collaboration used, with the size of the change being higher for the dimension related to the organisational setting (0.63) than for interpersonal relationships (0.47).

**Conclusions:**

Before and after the creation of the Bidasoa Integrated Healthcare Organisation, an improvement in the perceived degree of interprofessional collaboration between primary and secondary care levels was observed. This finding supports the benefit of a multilevel and multidimensional approach to integration, as in the described Bidasoa case.

**Discussion:**

Results on the two dimensions of the measure of interprofessional collaboration used seem to point to the longer time required for interpersonal relationships to change compared to the organisational setting.

## Introduction

Several regions in the world have implemented integrated health-care systems in order to achieve better coordination between organisations and professionals working in different places and levels of care. It is believed that integrated health care could reduce duplication, facilitate communication, support interprofessional collaboration and ultimately, improve patient care [1].

Integration of health services can be defined as a ‘process of bringing together common functions within and between organisations to solve common problems, developing a commitment to shared vision and goals and using common technologies and resources to achieve these goals’ [2]. Interprofessional collaboration in health care is a process by which professionals from different disciplines collaborate to provide an integrated and cohesive approach to patient care [3]. A systematic review revealed that interventions promoting interprofessional collaboration significantly improve patient outcomes and health-care professionals' practices [4].

The links between integration and interprofessional collaboration have been explored in the past [5–7], but little evidence exists on the effects of integrated health care organisations on collaborative practices between providers from different professions. In a study conducted in Sweden, integrated care was not associated with improved interprofessional collaboration [8]. However, other experiences report positive relationships between integrated health care and interprofessional collaboration [7,9]. Exploring how the creation of new integrated care structures can impact interprofessional collaboration is thus essential in order to support health-care policies and sustain change.

As part of a large-scale system, transformation through its ‘Strategy to tackle the challenge of chronicity in the Basque Country’ [10], the public Basque Health Service (Osakidetza) has undertaken, since 2009, several initiatives for health-care integration between different care levels. These initiatives search overcoming the fragmentation of providers and services, with the ultimate purpose of improving health-care outcomes and the patient's experience of ‘the journey through the system’ while at the same time optimising the use of resources by the public health system. Recognising the validity of the various definitions and practices of integrated care [11–13], the Basque Strategy for Chronicity envisages a diversity of forms and implementation strategies, according to the identification of context-specific local solutions by local clinical and managerial leaders.

Among the initiatives under development in Osakidetza, three main types of health-care integration practices can be distinguished [14]: (1) cases of organisational integration, with the creation of Integrated Delivery Organisations in line with Shortell et al's [15] ‘organized delivery system model’, which merge a hospital with the primary care settings of its area of influence under a common managerial structure; (2) shared care models between primary and hospital care for complex patients; and (3) integration of care processes, with the establishment of shared clinical pathways between primary and specialised care for specific diseases or speciality units. These last two types of integration forms can be found both under the same organisational structure (as is the case of the integrated delivery organisations) and between different organisations. Regarding the implementation strategies, in some cases, integration projects have initially originated from the top decision level and subsequently followed a top-down implementation process. This has typically been the case for the creation of the integrated delivery organisations. In other cases, health-care integration initiatives have emerged from the clinicians and other frontline health professionals through a bottom-up innovation process. In practice, the intricate nature of health systems results in these different types of integration models and innovation strategies to be found in combination across the Basque health service.

### Case under analysis

This article analyses for the development of the Bidasoa Integrated Healthcare Organisation, an initiative of organisational integration at the macro level, was initially promoted by the managerial leaders, who at the same time encouraged and supported the integration of the clinical processes between different care levels. In this sense, the approach to integration of the Bidasoa Integrated Healthcare Organisation's promoters responded to their vision about the need for a multifactorial approach to the improvement in the care model [10,16]. The Bidasoa Integrated Healthcare Organisation was the first example of organisational (or structural) integration developed in the Basque Health Service (Osakidetza). It was created in the Bidasoa health district, in the north-east of the Basque Country, in 2011, resulting from the merger of the three primary health centres and the secondary hospital of the district. It meant that these four previously separated and geographically close, healthcare settings were brought together under a single management structure and a common contracting and financial framework, which would jointly serve a population of about 85,000 people.

The creation of the Bidasoa Integrated Healthcare Organisation aimed to improve the care provided to the population. The care model being pursued would be characterised by its patient-centeredness, the continuity of care and a proactive approach to the care needs of the population being served, consistent with the definition of an ‘organized delivery system as a network of organizations that provides or arranges to provide a coordinated continuum of services to a defined population and is willing to be held clinically and fiscally accountable for the outcomes and the health status of the population served’ [15].

A key objective of the promoters of the Bidasoa Integrated Healthcare Organisation, considered as a necessary condition for the implementation of their pursued care model, has been fostering a change in the model of relationships between health professionals towards enhanced interprofessional communication and collaboration across care levels. This shift in the model of relationships has been driven by measures aimed at different components of integration and, namely, at [17] stimulating a cultural change, integrating the clinical system and developing a new shared governance structure. A first step in the creation of a cultural change was the elaboration and communication of a common strategic plan for the whole Integrated Healthcare Organisation (considering, therefore, both primary and specialised levels together) for years 2011–2013. The new organisational culture being envisaged should support communication between health professionals across units. With this purpose, a joint technical committee (with representatives from both primary and specialised care levels) was created as a body to promote clinicians participation and several joint clinical and health-care committees were set up on various topics including patient safety, pharmacy and clinical records, among others. As regards integration of the clinical system, progress was made towards the introduction of a shared electronic health record between primary and specialised care; integrated care pathways and clinical protocols were developed, and a continuity of care unit located at the hospital was established. The role of the continuity of care unit is to support the primary care teams in treating complex cases, as well as to improve the continuity of care for patients with multiple conditions across care levels. With regards to governance, the development of a shared leadership was promoted through the setting up of clinical management units and of a more transparent information system (through, for example, the use of a single management scoreboard for the whole Integrated Healthcare Organisation and the setting up of a joint board of hospital and primary health centres' directors) [16].

Complementing the measures taken to influence the model of relationships between professionals in the search for an improved model of health-care provision to the population, additional organisational innovations have also been introduced. These measures are primarily oriented towards developing a more proactive approach to care and include the introduction and use of predictive modelling for case finding and population health management. Predictive modelling allows estimating the future care requirements of each citizen, whose level of need is represented by a risk score in the electronic health record. Using these and other identification tools allows new ways of working and organising services in a proactive manner. At this initial stage, most efforts in the Bidasoa Integrated Healthcare Organisation have been concentrated on using these case-finding tools for a better management of multimorbid chronic patients (on whom are concentrated a high share of the costs of care, for example, 1% of the most resource-demanding patients in the public Basque Health Service - namely, complex polypathological patients - represents about 22% of the total health budget) [18], through the continuity of care unit. Other uses have been the identification of target patients to be followed under the agreed integrated care pathways.

## Objectives of the study

This study aims at assessing the change in the degree of interprofessional collaboration across two different levels of care, before and after the establishment of the Bidasoa Integrated Healthcare Organisation, based on the perceptions of two groups of health professionals (doctors and nurses). The underlying hypothesis was that the development of the integration project would lead to an improvement in the degree of collaboration between health professionals, compared to the baseline level, that is, before setting up the integrated delivery organisation.

## Theory and methods

To test the hypothesis of a change in the degree of interprofessional collaboration between care levels since the creation of the Integrated Healthcare Organisation, this study uses a questionnaire developed and validated elsewhere [19] that measures collaboration between clinical professionals at different levels of care, according to the opinion of the clinicians involved. It is composed of 10 items, four concerning personal relationships between professionals and the other six the organisational setting [19]. This questionnaire was based on the D'Amour framework [20–21], which identified 4 dimensions and 10 indicators in a process of interprofessional collaboration in health-care organisations (see Table 1).

## Methodology

For this study, a one-group pre-test/post-test pre-experimental design was adopted [22]. The perceived degree of collaboration was measured 7 months before establishing the Integrated Healthcare Organisation (May 2010) and 12 months after its creation (January 2012) among professionals (doctors and nurses) from the various health-care teams involved in the new Integrated Healthcare Organisation.

### Population and sample

To estimate the sample size required, an alpha of 0.05, a beta error of 0.05 (power of 95%) and a minimum detectable difference of 0.5 points before and after the creation of the Integrated Healthcare Organisation, with a standard deviation of 0.6 (taken from results in previous pilot studies [19]), were considered. This led to a minimum necessary sample size of 140 professionals. The total study population comprised 367 clinical professionals (doctors and nurses) working in the four health-care settings that form the Bidasoa Integrated Healthcare Organisation.

The sample selection was based on selective invitation to group sessions by the survey administrators. Both for pre-test and for post-test, group sessions of five–seven professionals were organised in each health-care setting. At primary care level, all doctors and nurses from the three health-care centers were invited to participate in the survey sessions. At specialised care level, clinicians (doctors and nurses) invited to take part in the survey belonged to a diverse range of the hospital units/services judged to be representative of those units with more potential for interaction with the primary care level. At the end of each session, health professionals were invited to participate in the survey on a voluntary basis and anonymously by completing a written questionnaire. At pre-test, the D′Amour's theoretical model of interprofessional collaboration [20] and the items of the survey were presented and explained to health professionals before their completion of the questionnaire. This was not considered necessary at post-test, given that by then, the D'Amour model of collaboration already had large diffusion among the participants.

### Data collection

The questionnaire used to collect the clinicians’ opinions assesses the degree of collaboration between health-care professionals working in different levels of care. Responses are given on a 5-point Likert scale, with 1 corresponding to the lowest degree of development of collaboration and 5 to the highest. The questionnaire used at post-test corresponds to the final Spanish version of a questionnaire validated elsewhere [19], whose English translation is included in the Appendix. The results from the validation of this questionnaire showed good initial validity and reliability in the context of the public Basque Health Service [19]. The factor analysis indicated the presence of two differentiated dimensions that together explained nearly 60% of the variance. The internal consistency analysis resulted in a Cronbach's alpha coefficient of 0.8. At pre-test, a preliminary version of this questionnaire was used. The main changes in the content and format of the questionnaire between pre-test and post-test were the following: (1) at pre-test, description of points 1, 3 and 5 of the Likert-scale (response options) were provided, while at post-test, description of points 2 and 4 were also added, (2) the wording of response options for each Likert point was made more uniform across different items in the post-test version (e.g. use of similar adjectives to describe the intensity of the same Likert point number across items), (3) the wordings in the questions that could imply value judgements and, therefore, risked biasing the responses that were eliminated between the pre-test version and the post-test version of the questionnaire.

At pre-test, information on the health-care setting (organisation and care level) of practice of the respondent was collected. At post-test, the questionnaire also collected sociodemographic data of respondents regarding age, sex and years of experience working in the same organisation and professional role (primary care doctor, primary care nurse, hospital specialist and hospital nurse).

### Statistical analysis

First, descriptive analysis was performed, using measures of central tendency and dispersion for continuous variables and percentages and frequencies for categorical variables. Subsequently, the degree of interprofessional collaboration was compared before and after the intervention with the Student's *t*-test for independent samples, setting the level of significance at 0.05. Prior to applying this test, the normality of the data was confirmed using a combination of statistical tests and plots. All the statistical analyses were performed using SPSS software (version 14.0). Missing data only represented a 0.9% of total data and were omitted from the analysis.

## Results

Both at pre-test and at post-test, all the professionals who participated in the group discussions (28 persons at pre-test and 118 at post-test) completed the questionnaire. The difference in the size of the pre-test and post-test cohorts, which can be justified by the greater diffusion of the integration project in the organisation at post-test, was taken into account in the estimation of the study's sample size. The final sample size obtained was of 146 observations.

Sociodemographic data were only compiled for the post-test sample. However, there was information about the care level of the respondent in both samples, which showed that the proportion of health professionals from hospital and primary care was equivalent in both groups (*χ* ^2^=0.28; *p*=0.596). The potential influence of belonging to a particular organisation on respondents' interprofessional collaboration scores (cluster effect) was checked by calculating the intraclass correlation coefficient. The intraclass correlation coefficient was 0.005, thus indicating that the respondents' scores were not influenced by the fact that they belonged to a specific health-care organisation.

In the post-test sample, the mean age of respondents was 45.54 years (SD: 10.12) and they had a mean of 3.41 years (SD: 1.48) of experience working in the same health-care organisation (this being the same hospital or the same primary care organisation). Well over half of the sample (79.6% of those that reported their sex) were women, which is slightly higher than the representation of women in the group of doctors and nurses in the total of the Basque health service (71.8%) [23]. This seems due to a higher share of women among the doctors in the sample (59% of doctors in the sample that reported their sex were women, compared to 48.1% of female doctors in the total of the Basque health service's staff).

**Figure 1.**
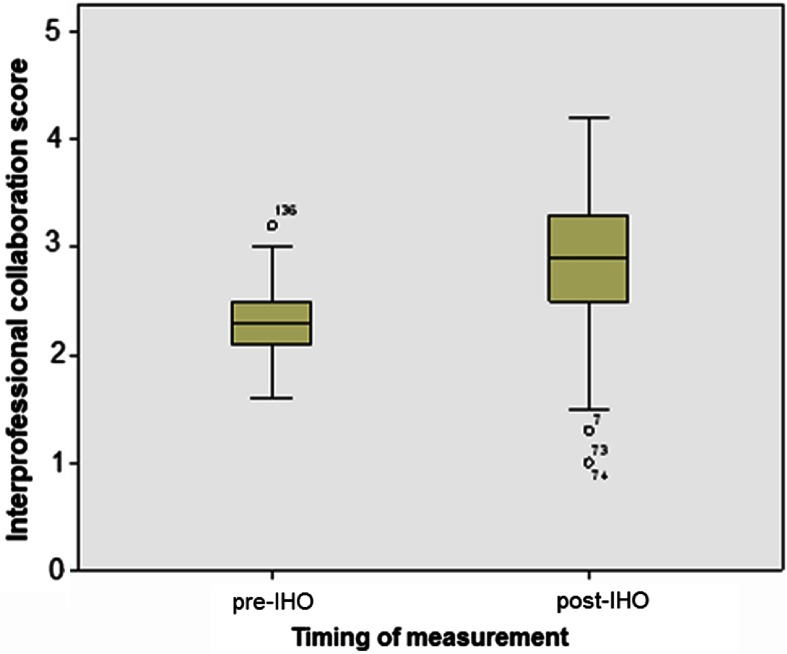
Box plot of the collaboration scores.

The mean scores for interprofessional collaboration before and after the intervention were 2.29 (SD: 0.37) and 2.86 (SD: 0.65), respectively. The Student's *t*-test indicated that this improvement in the perceived degree of collaboration by 0.57 points was statistically significant (*p*<0.001; CI 95%: 0.37–0.77), as can be observed in Table 2. Figure 1 is a box plot of the degree of collaboration before and after the intervention.

With respect to the interpersonal relationships and organisational setting dimensions, the analysis also showed that there were statistically significant differences between the two measurements, as shown in Table 2. The scores on interpersonal relationships before and after the intervention were 2.53 (SD: 0.51) and 3.00 (SD: 0.66), respectively, the difference being statistically significant with a *p* value of 0.001 (95% CI: 0.2–0.75). As for the organisational setting, the scores were 2.14 (SD: 0.49) and 2.77 (SD: 0.72) before and after the intervention, respectively, the Student's *t*-test again showing that the difference was significant (*p*=0.001; 95% CI: 0.39–0.87).

## Discussion

The degree of collaboration between health-care professionals (in this case, doctors and nurses) before the intervention had a mean score of 2.29. Personal relationships between professionals were rated better (2.53) than the organisational aspects (2.14). After the intervention, an improvement was observed in the perceived degree of collaboration between health professionals by 0.37 to 0.77 points. This finding supports the achievement of one of the main goals pursued by the establishment of the Bidasoa Integrated Healthcare Organisation, that is, the progress towards a collaborative model of relationships between professionals of different care levels, as measured according to the perception of the clinicians.

Health professionals reported a greater improvement in the assessment of the organisational aspects of collaboration (by 0.63 points) than in the aspects of personal relationships (by 0.47 points). From a theoretical point of view, D'Amour et al. [24] already pointed out that the intensity and implementation of the different factors determining the collaborative process can have a huge variation, which is influenced by the specific circumstances and contexts.

The fact that a more positive evolution was found as regards the organisational factors may be due to the fact that the measures put in place for developing the Integrated Healthcare Organisation mainly involved the introduction and strengthening of organisational structures. So emphasis was placed on the development of tools for formalising the interaction between different health professionals and units (clinical guidelines, committees, etc.), on the establishment of formal channels of communication and information exchange (continuity of care unit, shared medical record, intranet, etc.), as well as on the development of a shared leadership and governance structure (a common strategic plan and clinical management units). All of these measures, both those for the formalisation of interactions and those related to governance and leadership, are part of the organisational dimension of interprofessional collaboration [20]. The organisational dimension, such as it has been defined in this study, is key for understanding the dynamics of collaboration. Indeed, according to Philips et al [25], collaboration cannot be explained without consideration given to the existing organisational rules and norms as well as to the resources put in place.

Another aspect to be considered is that in a process of collaboration between organisations, the elements linked to interpersonal relationships require time to emerge and can pass through very different phases of development and consolidation. Indeed, some of the measures introduced in order to establish a shared vision and goals for both care levels, like the adoption of a common strategic plan, might take longer to be internalised by clinicians and, therefore, reflected in their responses to the questionnaire. Moreover, even if some measures aimed at enhancing mutual knowledge (such as the joint committees or the shared intranet) have been introduced, these might have still involved only a small share of the total number of clinicians. In addition, these interpersonal relationships will depend on the specific role played within the relationship, on the associated degree of uncertainty, on the expectations regarding the results of such relationship, and on the use of trust for conflict resolution [26]. Time does, therefore, seem fundamental in the development of all these factors, which might be a reason for the improvement in the assessment of the interpersonal aspects of collaboration to appear in a longer run.

The methodological limitations of this study include the fact that the questionnaire used for assessing the degree of collaboration was slightly changed between the surveys. Specifically, the wording of several items and response options was changed in order to establish a more uniform style among all items and response options of the questionnaire. Also, sociodemographic data of the respondents were not compiled in the first survey. It is thus not possible to verify if the two samples were equivalent. However, the proportions of professionals from hospital and from primary care were similar in both surveys. Given that the homogeneity of both samples (pre- and post-test) could only be tested on the variable ‘care level’, there could be other plausible hypotheses explaining the difference in the intensity of collaboration found between pre-test and post-test, besides the organisational integration project. Besides, the possibility of a ‘Hawthorne Effect’ affecting the perceptions of the staff involved in this integration project could not be dismissed. This effect might, however, be mitigated by the characteristics of the questionnaire being used [19,20], whose items focus on the determinants of interprofessional collaboration, several of which are likely to be considered by health professionals beyond their control, especially in the dimension related to the organisational setting, where the improvement found was higher. Given the methodological limitations of this analysis, the authors are cautious about the interpretation of the study results.

The integrated care network experience in the Basque Country is not unique as many countries support the integration of care in order to deal with the challenges and complexity of chronic health conditions [27–29]. However, the fact that this project is embedded in a global strategy [10] makes it difficult to generalise to other settings. It can, nonetheless, provide an exemplar of how interprofessional collaboration can be facilitated by structural changes and organisational support. Another limitation of this study is the fact that it only involved nurses and physicians, whereas other health-care providers also have a key role in interprofessional care teams. However, the limited role played by other types of clinical professionals in the specific case of the Basque Health Service precluded their inclusion in this study, which focuses on the collaboration across care levels (between primary and specialised care levels), as one of the main objectives of the Bidasoa integration project. However, future work should also consider how integrated care organisations can improve collaboration across health-care professions.

## Conclusion

The principal contribution of this study is the fact that it is the first to apply a valid instrument to assess changes in interprofessional collaboration between different care levels, following the establishment of an Integrated Delivery Organisation at the macro level, within the context of the new health-care policy for chronicity in the Basque Country.

The results show an improvement in the perceived degree of interprofessional collaboration between primary and secondary care levels before and after the creation of the Bidasoa Integrated Healthcare Organisation. Data in this study also indicate that the effect of integration on interprofessional collaboration would not be influenced by intraorganisational factors, given the minimal intraclass correlation coefficient found. Nevertheless, this finding needs to be confirmed in other contexts and with larger samples of organisations.

In explaining the positive relationship between the creation of the integrated delivery organisation and the degree of interprofessional collaboration between health-care professionals from different levels of care, it is important to note that the approach to integration followed in the Bidasoa Integrated Healthcare Organisation combines the organisational integration at the macro-level, with other initiatives for integration of the clinical processes. It is also worth noting the explicit consideration of the role of the cultural dimension in the integration process and the active approach to influence it. The case of the Bidasoa Integrated Healthcare Organisation, therefore, seems to confirm the benefit of a multilevel and multidimensional approach to integration, as envisioned by its promoters.

## Reviewers

**Íngrid Bullich Marín**, Technical Health Officer, Social Health Care Plan, Ministry of Health, Catalonia, Spain

**Bernadette Hannigan**, Professor, Chief Scientific Advisor - Department of Health, Social Services and Public Safety, Northern Ireland

**Dominique Tremblay**, Assistant Professor, School of Nursing, Faculty of Medicine and Health Sciences, University of Sherbrooke, Canada

## Figures and Tables

**Table 1.**
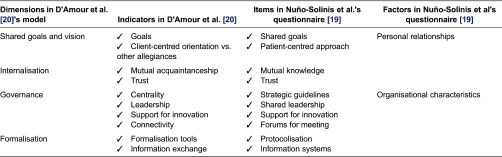
Relationships between the structure of the theoretical framework and the study questionnaire.

**Table 2.**
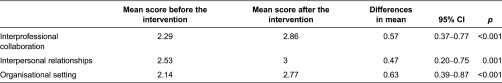
Mean collaboration scores before and after the intervention and the differences in the mean.

**Appendix:**
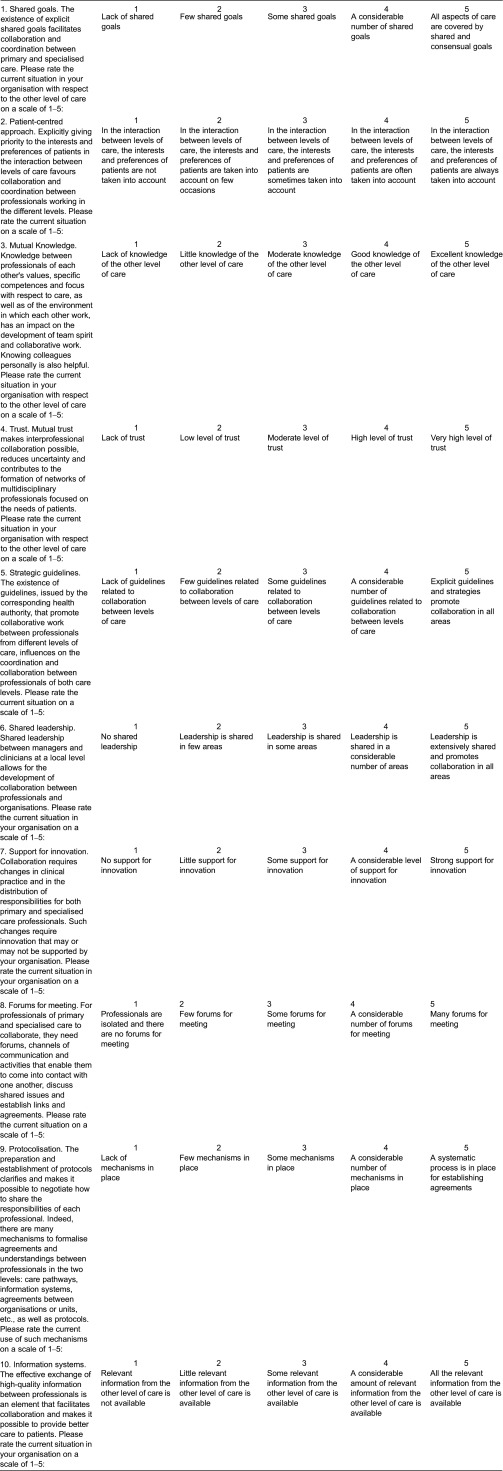
Questionnaire to assess interprofessional collaboration between two different care levels – English translation.
